# *Pseudomonas putida* Chemotactic Efficiency toward Naphthalene at a NAPL–Water Interface Decreased under Increasing Shear Flow

**DOI:** 10.1021/acs.est.5c15041

**Published:** 2026-01-29

**Authors:** Beibei Gao, Rhea Braun, Derek Wu, Roseanne M. Ford

**Affiliations:** Department of Chemical Engineering, 2358 University of Virginia, Charlottesville, Virginia 22903, United States

**Keywords:** Chemotaxis, Reversal frequency, Shear flow, Agent-Based model (ABM), Continuum model, Nonaqueous phase liquid (NAPL), Microfluidics

## Abstract

Chemotactic bacteria have the potential to enhance the bioremediation of nonaqueous phase liquid (NAPL) pollutants by preferentially migrating toward contaminant sources. Although groundwater flow has been shown to influence bacterial chemotaxis, its quantitative influence on intrinsic motility parameters governing chemotactic strategies remains unresolved. Using a T-shaped microfluidic device, mimicking a NAPL droplet trapped within a pore throat, we show that chemotactic bacteria exhibited greater retention near the NAPL interface at low fluid velocities (0.5 m/d and 1 m/d; corresponding wall shear rates of 0.58 and 1.16 s^–1^, respectively), while both population density and accumulating area declined at higher velocities. Continuum-level simulations of bacterial transport indicated a reduction in the chemotactic sensitivity coefficient *χ*_o_ by an order of magnitude at flow velocities above 5 m/d (5.78 s^–1^). Trajectory analysis of bacteria from videomicroscopy revealed increasing alignment of bacterial motion with the flow direction as the fluid velocity increased. We conducted agent-based model simulations to further demonstrate that flow-induced suppression of reversal frequency reduced chemotactic efficiency in *Pseudomonas putida*. This work demonstrated a framework that integrates experimental and modeling approaches at both population and individual scales to investigate mechanisms underlying bacterial transport phenomena.

## Graphical abstract

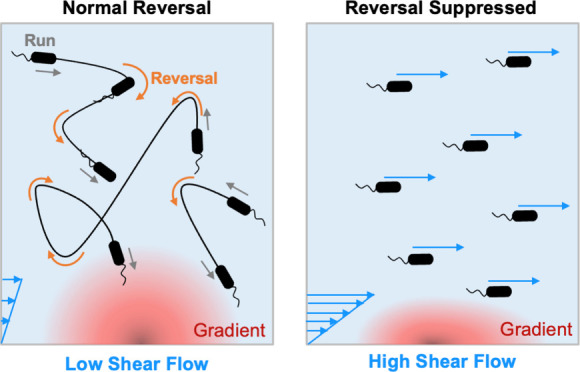

## Introduction

Chemotaxis-aided bioremediation leverages microbial metabolism and the ability of bacterial populations to migrate directionally in response to chemical gradients, offering a cost-effective strategy for pollution mitigation. Chemotaxis is promising for addressing persistent soil contaminants such as nonaqueous phase liquids (NAPLs), which are often trapped in hydraulically inaccessible regions following aggressive remedial treatments like flooding and flushing.^1^ Some soil bacteria (e.g., *Pseudomonas putida*) are chemotactic toward NAPL pollutants including naphthalene, hexadecane, benzene, and trichloroethylene.^2^ These chemotactic bacteria can locate and aggregate at NAPL surfaces,^3–5^ initiating metabolic activity and biodegradation of NAPLs. In subsurface environments, fluid flow is a prevalent feature of the bacterial habitat, transporting bacteria through porous media, shaping chemical landscapes, and altering bacterial swimming.

Bacterial motility involves alternating between straight runs and reorientation events driven by flagellar motors. However, fluid flow imposes shear stresses on bacteria that can alter their swimming patterns and suppress chemotactic responses.^6^ Hydrodynamic forces influence bacterial motility through several mechanisms, including Jeffery orbits^7^ and rheotaxis.^8–10^ Shear flow can also disrupt bacterial orientation; for example, rod-shaped bacteria rotate less frequently when aligned with the flow and more rapidly when oriented transversely,^11^ leading to preferential alignment along the flow and so-called shear trapping in high-shear, low-velocity zones near surfaces.^12^ Yang et al.^13^ and Liu et al.^14^ found that shear flow can interfere with flagellar dynamics; in Liu et al.^14^ flow-induced bundling promotes longer runs and shorter tumbles in *Escherichia coli*.

When exposed to chemical gradients, bacteria can bias their motion by extending run durations in favorable directions (e.g., up a chemoattractant gradient), which is a key feature of chemotaxis. Increasing flow velocities have been shown to impair chemotactic efficiency,^15–18^ including in *Pseudomonas putida* (*P. putida*). *P. putida* swim in straight runs interrupted by sharp reversals to reorient its trajectory.^19,20,20,21^ The mechanisms underlying the reduced chemotaxis in *P. putida* under shear flow remain incompletely understood, yet elucidating them is crucial for improving NAPL bioremediation.

This study aimed to investigate the influence of fluid flow on the chemotaxis and motility of *Pseudomonas putida*. Chemotactic and nonchemotactic *P. putida* strains were exposed to chemoattractant gradients originating from a flat NAPL–water interface, mimicking an NAPL source trapped within a pore throat in porous media, under varying flow velocities in a T-shaped microfluidic device. Bacterial and chemoattractant distributions in the experimental systems were modeled using a modified advection-dispersion transport model. To further examine how fluid flow alters bacterial swimming behaviors, we implemented an agent-based model (ABM) to simulate modifications in run and reversal dynamics under flow, using the identical system geometry as in the experiments. ABM results were compared with experimental observations and used to rationalize parameter adjustment in the continuum model, enhancing its applicability to field-scale bioremediation scenarios. This work sheds light on the mechanism by which fluid flow interferes with bacterial runs and reorientations, ultimately leading to reduced chemotactic performance. More broadly, our study establishes a multiscale modeling framework that integrates individual- and population-level approaches to explore how fluid dynamics shape bacterial behavior in complex environments.

## Materials and Methods

### Preparation of Bacterial Culture

Two motile bacterial strains were used in this study: *Pseudomonas putida* G7 (*Pp*G7, chemotactic to naphthalene) and *Pseudomonas putida* G7 Y1 (*Pp*G7 Y1, a *nah*Y::Km mutant and nonchemotactic to naphthalene).^22^ Cultures of *Pp*G7 and *Pp*G7 Y1 were prepared following the protocol in Grimm and Harwood^22^ with minor modifications as described previously in Gao et al. (2023).^23^ Prior to transport experiments, bacterial motility was visually checked under a 100× oil lens with a Zeiss microscope (Zeiss KF2, NY). Bacterial chemotaxis to naphthalene was verified in a chemotaxis drop assay, following the protocol in Grimm and Harwood.^22^

### Micromodel Design and Operation

In bacterial transport experiments, we used a microfluidic device following the design from Wang et al.^16^ Chip design, fabrication, and operation are described in detail there. We obtained a silicon wafer with a raised T structure from the nanoFAB facility at the University of Alberta, Canada. Polydimethylsiloxane (PDMS, Fisher Scientific, PA) was mixed with its curing agent at a weight ratio of 10:1 and degassed prior to pouring onto silicon water. The mixture and silicon wafer were baked in an Isotemp Oven (Fisher Scientific, KS) at 90 °C for an hour. Then the PDMS with the duplicate T-shape channel was peeled from the wafer and sealed with a cover glass after plasma treatment (Henniker HPT100, UK) for 45 s.

The experimental setup and T-shaped microfluidic chip are illustrated in Figure 1a. In brief, bacteria suspended in a 1:10 dilution of random motility buffer at neutral pH (∼7) were introduced into the macrochannel via a 100 μL syringe loaded on a syringe pump (PHD 2000 Infusion, Harvard Apparatus, Holliston, MA). Mineral oil (J.T. Baker, USA) was pushed into the side capillary slowly via another 100 μL syringe until the NAPL–water interface was stabilized at the edge of the macrochannel, as shown in Figure 1b. Prior to the introduction of bacterial suspension or oil, deionized water was flushed at a high velocity and pressure throughout the T-shaped chamber to remove any air bubbles. The chip dimensions were 20 μm in depth with lengths and widths of 5 cm × 2.5 mm and 1 cm × 50 μm for the macrochannel and capillary, respectively.

**Figure 1:**
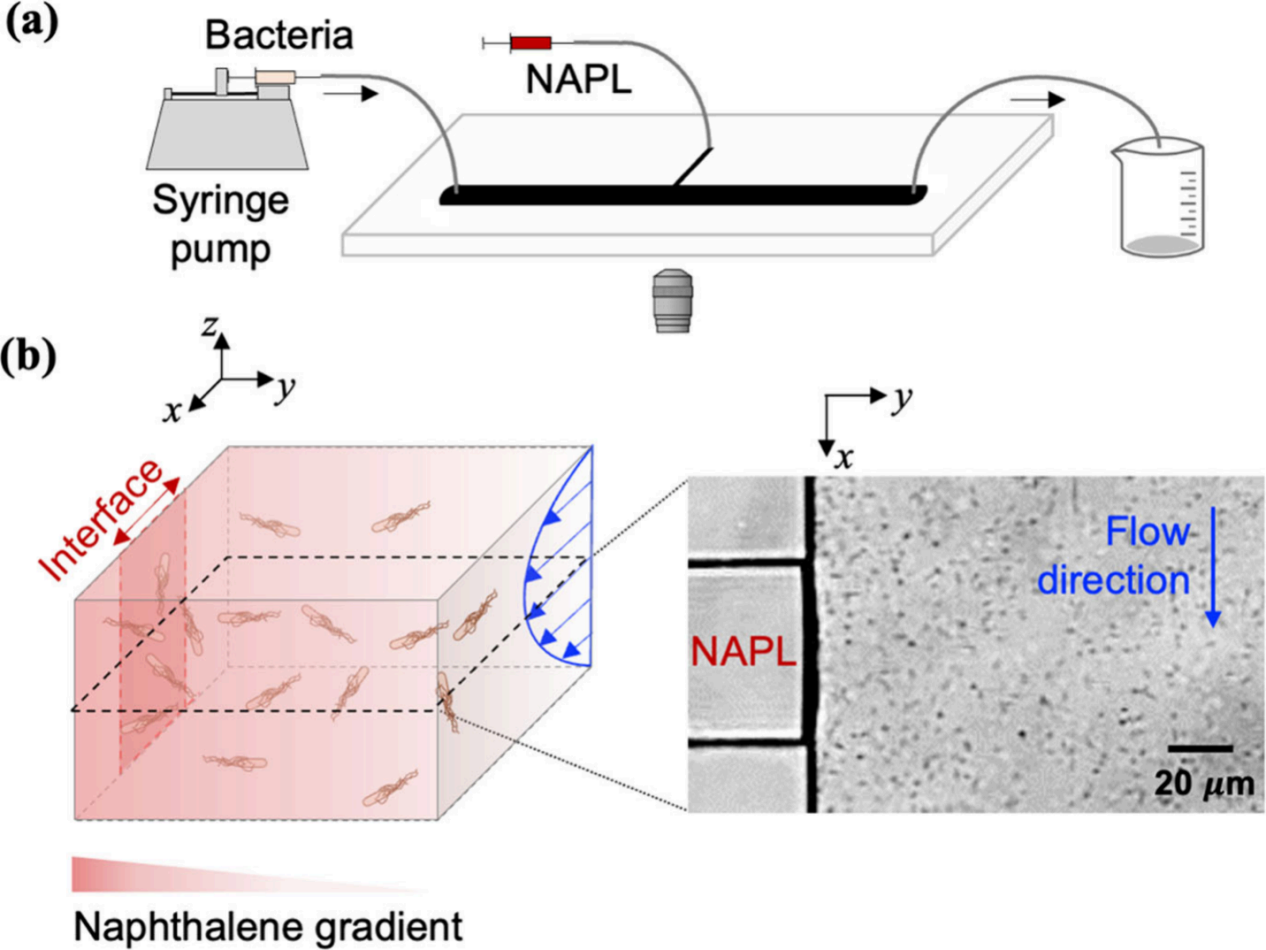
T-shaped microfluidic device. (a) Schematic view of experimental setup with inverted microscopy and design of the microfluidic chamber. The T-shaped chamber is 20 μm in depth and consists of a macrochannel and side capillary. The macrochannel, flow path for bacterial suspension, is 5 cm long and 2.5 mm wide, and the side capillary, a reservoir for NAPL, is 1 cm long and 50 μm wide. (b) A three-dimensional illustration of bacteria, naphthalene concentration, and fluid velocity profile near the NAPL–water interface. Bacteria were imaged at midchannel height in the vicinity of a NAPL–water interface.

### Bacterial Transport Experiment

To investigate the influence of shear flow on bacterial chemotaxis, a mixture of mineral oil containing 33 g/L naphthalene (chemoattractant for *Pp*G7) was introduced into the capillary. Naphthalene concentration in the aqueous phase at equilibrium with mineral oil containing 33 g/L naphthalene was measured to be 0.25 mol/m^3^ by a gas chromatograph mass spectrometer GCMS-QP2020 NX (Shimadzu, Germany) (details in the Supporting Information). Mineral oil is highly viscous (80–100 cP at room temperature), which helped to stabilize the NAPL–water interface. Mineral oil also prevented hydrocarbons from dissolving into PDMS. As controls, *Pp*G7 and nonchemotactic *Pp*G7 Y1 were injected into the chamber in the absence and presence of naphthalene, respectively, at fluid velocities ranging from 0.5 to 10 m/d (wall shear rate from 0.58 to 11.57 s^–1^). Each combination of bacteria and the NAPL mixture was repeated in triplicate. Bacteria were continuously flowed through the chamber at uniform density and imaged at the midheight plane near the NAPL–water interface, as illustrated in Figure 1b. In each run, fluid velocity was initially set at 10 m/day and then reduced to 5, 1, and 0.5 m/d. Images were taken at 10 min after adjusting the pump rate, by which time simulations indicated that flow velocity, bacteria, and naphthalene concentration profiles had reached steady state. All experiments were conducted in a room with a controlled temperature (22 °C).

### Data Acquisition and Processing

A wide-field microscope (Olympus IX-70, FL) with a phase ring No. 1, 20×/0.40 lens and a CCD camera was used to record bacterial distributions in the regions of interest near the NAPL–water interface. 100 images were taken at the NAPL–water interface consecutively at a time interval of 50 ms at each fluid velocity. Image sequences were imported into ImageJ^24^ as stacks, and the average image of each stack was subtracted to remove background noise. The resulting images were binarized to generate processed temporal series, which were used for quantitative analysis of bacterial distributions at different flow velocities. The processed images were further analyzed to determine the *y*-component of the bacterial swimming velocity. Using TrackMate,^25^ individual bacterial positions were tracked across sequential frames, and their velocities perpendicular to the flow direction were calculated.

### Continuum Model

A two-dimensional mathematical model was used to simulate chemoattractant and bacterial distributions in the *x-y* plane of the macrochannel. In bacterial transport experiments, a suspension of bacteria in a 10% random motility buffer (without additional nutrients) was introduced into the macrochannel. We assumed no proliferation or loss of bacteria and no consumption of naphthalene during the 1-h experiment. Therefore, the governing equation for chemoattractant naphthalene was reduced to the following convection-diffusion equation,

1 $$\frac{\partial a}{\partial t}=D_{a}\nabla^{2}a-v_{f}\cdot \nabla a$$

where *a* is naphthalene concentration in the aqueous phase (ML^–3^), *t* is time (T), *D*_a_ is the diffusion coefficient of naphthalene in water (L^2^ T^–1^), and ***v*_*f*_** is the fluid velocity (LT^–1^). We assumed that the naphthalene concentration on the aqueous side of the NALP–water interface was at its equilibrium concentration *a*_0_ = 0.25 mol/m^3^ and there was no mass flux at the microfluidic wall boundaries. Initially, naphthalene concentration was zero in the macrochannel.

The governing equation for chemotactic bacteria was a modified convection-diffusion equation with chemotactic velocity added in the convection term,

$$\frac{\partial b}{\partial t}=D_{b}\nabla^{2}b-\left(v_{f}+V_{C}\right)\cdot \nabla b$$ 2

where *b* is bacterial concentration in the aqueous phase (ML^–3^), *D*_b_ is the bacteria motility coefficient (L^2^ T^–1^), and **V**_**C**_ is the chemotactic velocity of the bacterial population (LT^–1^) defined as,^26,27^

3 $$V_{C}=\frac{2v}{3}\;\tanh\left[\frac{\chi_{o}}{2v}\frac{K_{C}}{{\left(K_{C}+a\right)}^{2}}\nabla a\right]$$

where *v* is bacterial swimming speed (LT^–1^), *χ*_o_ is chemotactic sensitivity coefficient of bacteria (L^2^ T^–1^) to naphthalene, and *K*_C_ is the chemotactic receptor constant (ML^–3^). The chemotactic velocity (**V**_**C**_) was set to zero for nonchemotactic bacteria or scenarios without chemoattractant. A bacterial suspension was introduced into the macrochannel at a fluid velocity of *v*_f_, and no mass flux of bacteria was applied to the NAPL–water interface and microfluidic walls.

COMSOL Multiphysics Version 5.6 simulation software (or COMSOL) was used to solve the partial differential equations for the chemoattractant and bacteria. Initially, there was no chemoattractant or bacteria in the macrochannel. Bacteria suspensions were introduced at the inlet at velocities ranging from 0.5 to 10 m/d. No-slip boundary conditions were applied to all walls, except the inlet and outlet. Naphthalene concentration at the NAPL-water interface was fixed at *a*_0_ = 0.25 mol/m^3^. Other parameters used in our simulation included: fluid velocity *v*_f_ = 0.5–10 m/d, naphthalene diffusion coefficient in water *D*_a_ = 7.5 × 10^–6^ cm^2^/s,^28^ bacterial motility coefficient *D*_b_ = 3.2 × 10^–6^ cm^2^/s,^16^ bacterial swimming speed *v* = 49 μm/s,^29^ chemotactic receptor constant *K*_C_ = 0.016 mol/m,^3,29^ and chemotactic sensitivity coefficient *χ*_o_ = 7.2 × 10^–5^ cm^2^/s at 0.5 m/d.^29^ Details about model implementation in COMSOL and parameters can be found in the Supporting Information.

### Agent-Based Models

Agent-Based Models (ABMs) are operated on a discrete grid with a specified number of movable “agents”, which are the individual actors that have defined variables and follow specific rules. In our ABM implementation, each bacterial agent followed rules that mimic the run-reversal swimming pattern of *Pseudomonas putida*, capturing realistic population motility behavior. Each agent’s motion was governed by swimming speed (*v*), baseline reversal frequency under no stimulus (*p*_0_), turn angle distribution^19^ and chemoeffector parameters (σ, *N*_T_, and *K*_C_). Under chemotactic conditions, *P. putida* agents change their frequency of reversal in response to temporal changes in local chemoeffector concentrations (*a*). Rivero et al. (1989)^26^ related the reorientation frequency *p*_*t*_^+^ during chemotaxis (T^–1^) to the chemoeffector gradients,

4 $$-\log\left(\frac{p_{t}^{+}}{p_{0}}\right)=\sigma \frac{dN_{b}}{da}\left(\frac{da}{dt}\pm v\nabla a\right)=\sigma \frac{dN_{b}}{da}\frac{Da}{Dt}$$

with

5 $$\frac{dN_{b}}{da}=N_{T}\frac{K_{C}}{{\left(K_{C}+a\right)}^{2}}$$

where σ is the sensitivity of the flagella reversal signal to the change in bound receptors, *N*_b_ is the concentration of bound receptors on the cell surface, *N*_T_ is the total number of chemotaxis receptors, *K*_C_ is the chemotactic receptor constant and *Da*/*Dt* is the chemoattractant material derivative. From eqs 4 and 5, we obtained the reversal probability of a cell undergoing chemotaxis as,

6 $$\log\left(\frac{p_{t}^{+}}{p_{0}}\right)=-\sigma^{*}\frac{K_{C}}{{\left(K_{C}+a\right)}^{2}}\frac{Da}{Dt}$$

where σ* is the effective single-cell chemotactic sensitivity, σ* = *σN*_T_. Rivero et al. (1989)^26^ related σ to the population-scale continuum chemotaxis sensitivity coefficient *χ*_o_ in eq 3 by *χ*_o_ = *σN*_*T*_ *v* ^2^. In our ABM model for *P. putida* chemotaxis toward naphthalene, the chemotactic parameter σ* was determined to be 4 s using the relation *χ*_o_ = σ* *v* ^2^. Values of *χ*_o_ = 7.2 × 10^–5^ cm^2^/s and *K*_C_ = 0.016 mM were from the literature.^29^

Our Agent-Based Model was constructed using the modeling platform NetLogo,^30^ which consisted of a 400 μm × 800 μm two-dimensional rectangular space as shown in Figure 2a that stored information related to naphthalene distribution and flow velocity. We imported naphthalene concentrations predicted from COMSOL solutions of eq 1 into the *x*-*y* plane, as shown by the blue color in Figure 2a. Figure 2b shows the logic loop flowchart used in our model. Initially, 4000 bacteria were created and randomly distributed within the simulation space. At each time step, agents calculated the reversal probability based on the naphthalene concentration and gradient at their patch. If the bacteria agents reversed, they selected a new direction for their movement based on a known distribution of turn angles^19,31,32^ and selected a new running speed at random from a normal distribution.^19,32^ If the bacteria decided to run, they moved forward based on their running speed adjusted by the time step. The swimming velocity of simulated *P. putida* was 44 μm/s, and the baseline reversal frequency was 0.5 s^–1^ in static liquid.^32,33^ To account for the impact of the fluid velocity, bacteria would move a certain distance downstream, depending on the local fluid velocity at their assigned vertical position. The time step was set to 0.1 s, matching the time associated with an reorientation event as reported in the literature.^33^ Figure 2c shows representative simulated bacterial trajectories in NetLogo in the presence of a naphthalene gradient over a period of 20 s.

**Figure 2:**
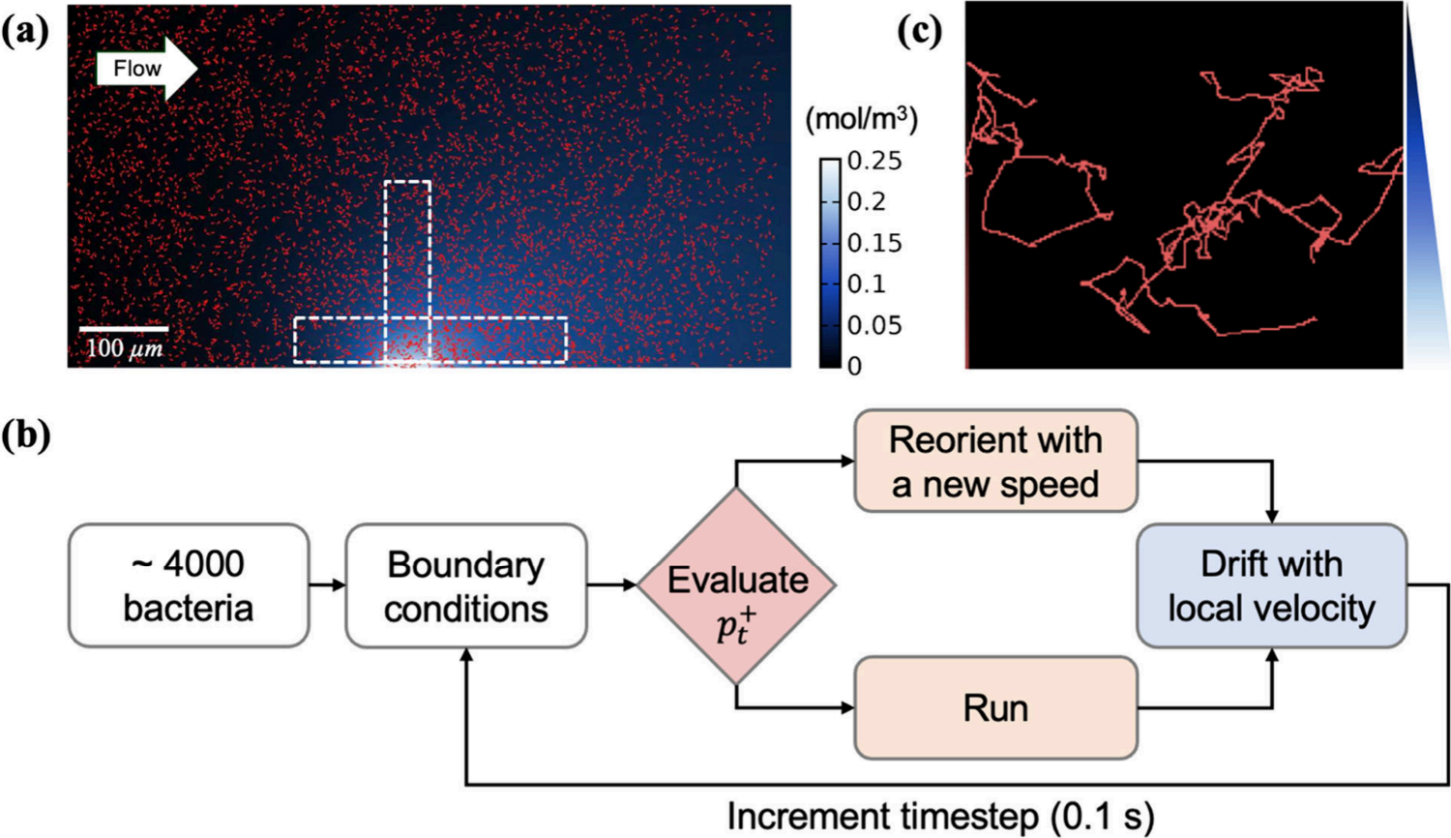
(a) Bacterial agents (red ellipsoids) simulated in the ABM platform for a 400 μm × 800 μm grid. Naphthalene concentration is represented in shades of blue, corresponding to the color bar. Fluid flow with an average velocity of 0.5 m/day was introduced in the direction parallel to the NAPL–water interface as indicated by the arrow in the top left corner. Regions of interest corresponding to experimental analyses are depicted by dashed white lines. (b) Logic loop flowchart for the ABM model. (c) A few bacterial trajectories (red lines) simulated over a 20-s time period within a naphthalene gradient.

The fluid velocity was stored as an agent parameter following Poiseuille flow at the average fluid velocity. To implement the fluid flow boundary conditions, bacteria were continuously spawned in the leftmost region of the world and continuously removed from the rightmost region of the world such that the concentration of bacterial agents in those regions was kept constant and equal to a baseline value. Bacteria agents were reflected from the surface at a random angle if they collided with the grid boundary. At the end of loop, bacterial locations in the region of interest (enclosed by dashed white line in Figure 2a) were exported to compare with experimental observations and continuum model simulations. Table 1 compares the bacterial parameters used in the continuum and Agent-Based Models.

**Table 1:** Comparison of Parameters between the Continuum and Agent-Based Models

parameters	continuum model	agent-based model
Swimming speed *v*	49 μm/s	44 μm/s
Chemotaxis receptor constant, *K*_C_	0.016 mM	0.016 mM
Chemotactic sensitivity coefficient (at 0.5 m/d), *χ*_o_	7.2 × 10^–5^ cm^2^/s	-
Bacterial diffusion coefficient, *D*_b_	3.2 × 10^–6^ cm^2^/s	-
effective single-cell chemotactic sensitivity, σ^a^	-	4 s^a^
Reversal frequency (at 0.5 m/d), *p*_0_	-	0.5 s^–1^

a Calculated from *χ*_*o*_ = σ* *v* ^2^, *χ*_*o*_ = 7.2 × 10^–5^ cm^2^/s.

## Results and Discussion

### Bacterial Chemotaxis to a NAPL–Water Interface

In bacterial transport experiments, bacteria (chemotactic *Pp*G7 or nonchemotactic *Pp*G7 Y1) were introduced into the macrochannel for a range of fluid velocities (0.5–10 m/d), corresponding to a shear rate of 0.58 to 11.57 s^–1^. The side capillary was filled with either a mixture of chemoattractant naphthalene and mineral oil or mineral oil only. To investigate the influence of fluid flow on bacterial chemotaxis, we examined bacterial density near the NAPL–water interface in the directions perpendicular and parallel to the flow, respectively, as shown by the dashed white boxes in Figure 3a, left and right panels. In the scenario where the chemoattractant was dissolved in NAPL, the chemoattractant was released from the side capillary into the ambient aqueous phase; therefore, the vicinity of the NAPL–water interface was featured with naphthalene gradients (Figure 3b). At a higher average fluid velocity (e.g., 10 m/d in Figure 3b), naphthalene gradients were steep and confined to a smaller region. Figure 3c shows normalized bacterial density within a distance of 0–200 μm from the NAPL–water interface. In the presence of chemoattractant naphthalene (the left plot in Figure 3c), chemotactic bacteria *Pp*G7 accumulated to a greater extent within 50 μm from the NAPL–water interface as their densities were greater than unity, especially at 0.5 and 1 m/d corresponding to open circles and triangles, respectively. This accumulation was attributed to a chemotactic response to naphthalene as bacterial densities in the control cases (i) in the absence of naphthalene (Figure 3c center) and (ii) for nonchemotactic *Pp*G7 (Figure 3c right) remained near a normalized value of one. At an average fluid velocity of 0.5 m/d, chemotactic *Pp*G7 detected the presence of naphthalene and swam up naphthalene gradients against fluid flow, thereby accumulating near the NAPL–water interface and reaching a normalized density around 1.3. The advantage of chemotaxis in directing bacteria to the chemoattractant source was reduced at 1 m/d and disappeared at the higher velocities of 5 and 10 m/d. Our experiments also revealed that the increase in fluid velocity reduced the spatial extent of bacterial accumulation (Figure 3d). At 0.5 m/day, bacteria accumulated from 100 μm upstream to 100 μm downstream of the 50 μm interface (i.e., spanning −100 to 150 μm along the *x* axis). When the fluid velocity increased to 1 m/day, bacterial accumulation was confined to a smaller area near the interface within 25–75 μm along the *x* axis. At higher fluid velocities of 5 and 10 m/d no accumulation was observed. For the control cases, densities of *Pp*G7 in the absence of naphthalene and nonchemotactic bacteria *Pp*G7 Y1 along the flow direction remained near unity for all fluid velocities, as shown in Figure 3d.

**Figure 3:**
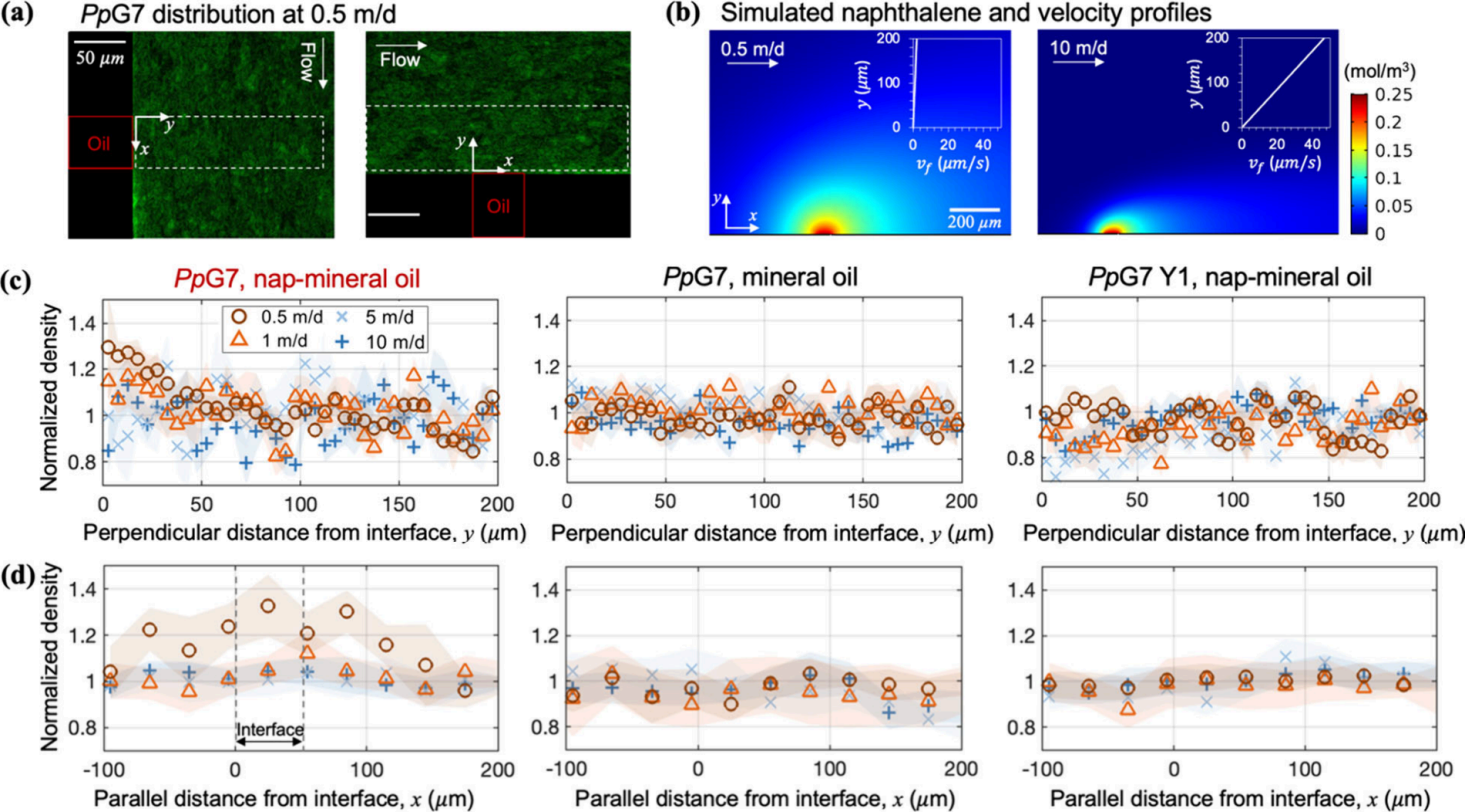
Bacterial, naphthalene, and fluid velocity distributions near the NAPL–water interface. (a) Distribution of *Pp*G7 in the presence of naphthalene at 0.5 m/day near the NAPL/water interface in the T-shaped microfluidic device. Dashed white boxes represent regions of interest for density quantification that are shown in (c) and (d). Panel (b) compares simulated naphthalene distributions at 0.5 and 10 m/d, together with the corresponding fluid velocity profiles (inset plots). Panels (c) and (d) are normalized density of chemotactic bacteria *Pp*G7 with naphthalene dissolved in mineral oil inside the capillary, *Pp*G7 with mineral oil only, and nonchemotactic bacteria *Pp*G7 Y1 with naphthalene dissolved in mineral oil. In (c) or (d), bacterial densities were averaged along the *x* or *y* direction and plotted against the distance in *y* or *x*, respectively. Standard deviation (shaded region) was calculated among three replicates.

### Shear Flow Reduces Apparent Chemotactic Sensitivity Coefficient

To investigate the influence of fluid flow on *Pp*G7’s chemotactic response toward naphthalene, we numerically solved a convection-diffusion equation with an added chemotactic velocity (eqs 2–3) to simulate the distribution of a bacterial population near a NAPL–water interface. Simulated bacterial distributions at varying fluid velocities (solid lines) are overlaid onto experimental data (symbols) in Figure 4. Parameters related to intrinsic properties of *Pp*G7 (swimming speed *v*, diffusion coefficient *D*_b_ and chemotactic receptor constant *K*_C_) were obtained from the literature.^16,29,34^ The only fitting parameter for bacterial transport was the chemotactic sensitivity coefficient, χ_o_, which reflects the strength of the chemotactic response. Simulation results suggest that the increase of fluid velocity systematically reduced the apparent χ_o_ value, as shown in Figure 4. Specifically, at a flow rate of 0.5 m/d, a good match of bacterial distributions in both the vertical and parallel directions to fluid flow between experiments and simulations yielded a value of *χ*_o_ = 7.2 × 10^–5^ cm^2^/s, consistent with the value reported by Marx and Aitken.^29^ When the fluid velocity increased to 1 m/d, the apparent χ_o_ value was reduced by 50%, suggesting reduced chemotactic efficiency under flow. At fluid velocities greater than 5 m/d, χ_o_ decreased by at least 1 order of magnitude, which indicates that bacterial transport was likely dominated by fluid convection. At flow velocities of 1, 5, and 10 m/d, bacteria were exposed to the interfacial region for approximately 4, 0.8, and 0.4 s, respectively, which limited the time available for bacteria to fully express biased chemotactic migration toward the interface. As a result, bacterial accumulation was likely diminished, reflecting reduced chemotactic efficiency (χ_o_) despite the presence of chemical gradients.

**Figure 4:**
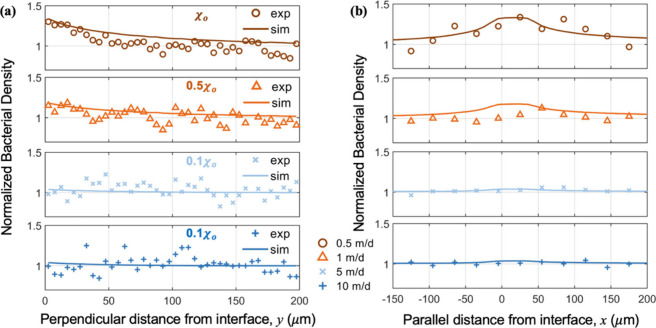
Chemotactic response of *Pp*G7 toward naphthalene at the NAPL–water interface for fluid velocities varying from 0.5 to 10 m/d along the (a) *y* and (b) *x* directions. Symbols and solid lines are experimental data and simulated results, respectively. Chemotactic sensitivity coefficient, *χ*_o_ = 7.2 × 10^–5^ cm^2^/s.

### Shear Flow Alters Bacterial Swimming Properties

In the absence of a chemoattractant, *P. putida* G7 bacteria trajectories showed an increasing alignment with the fluid flow direction as the fluid velocity increased from 1 to 5 m/d, as shown in Figure 5a. *P. putida* swimming speed reported as 44–49 μm/s,^29,33^ equivalent to 3.8 m/d, falls within this fluid velocity range. To evaluate the influence of fluid flow on bacterial swimming speed, especially in the direction perpendicular to flow, we extracted the *y*-component of swimming velocity using TrackMate^25^ and plotted the distributions as violin plots (Figure 5b). The width of each violin represents the proportion of bacteria at a given *y*-component velocity. At 0.5 m/day, the diamond-shaped violin plot indicates that most bacteria swam near the average *y* velocity of 10.1 μm/s. As fluid velocity increased, the violin shape broadened at lower velocities and developed longer upper tails, indicating that bacterial swimming in the *y* direction became increasingly skewed toward lower speeds. The average *y*-component velocity decreased from 10.1 μm/s to 8.5 μm/s to 5.0 μm/s at 0.5 m/d, 1 m/d, and 5 m/d, respectively. This decrease supports the observation that bacterial trajectories were more aligned with the flow direction at a higher flow velocity, suggesting that altered swimming behaviors may contribute to the reduced chemotactic response observed in Figure 4.

**Figure 5:**
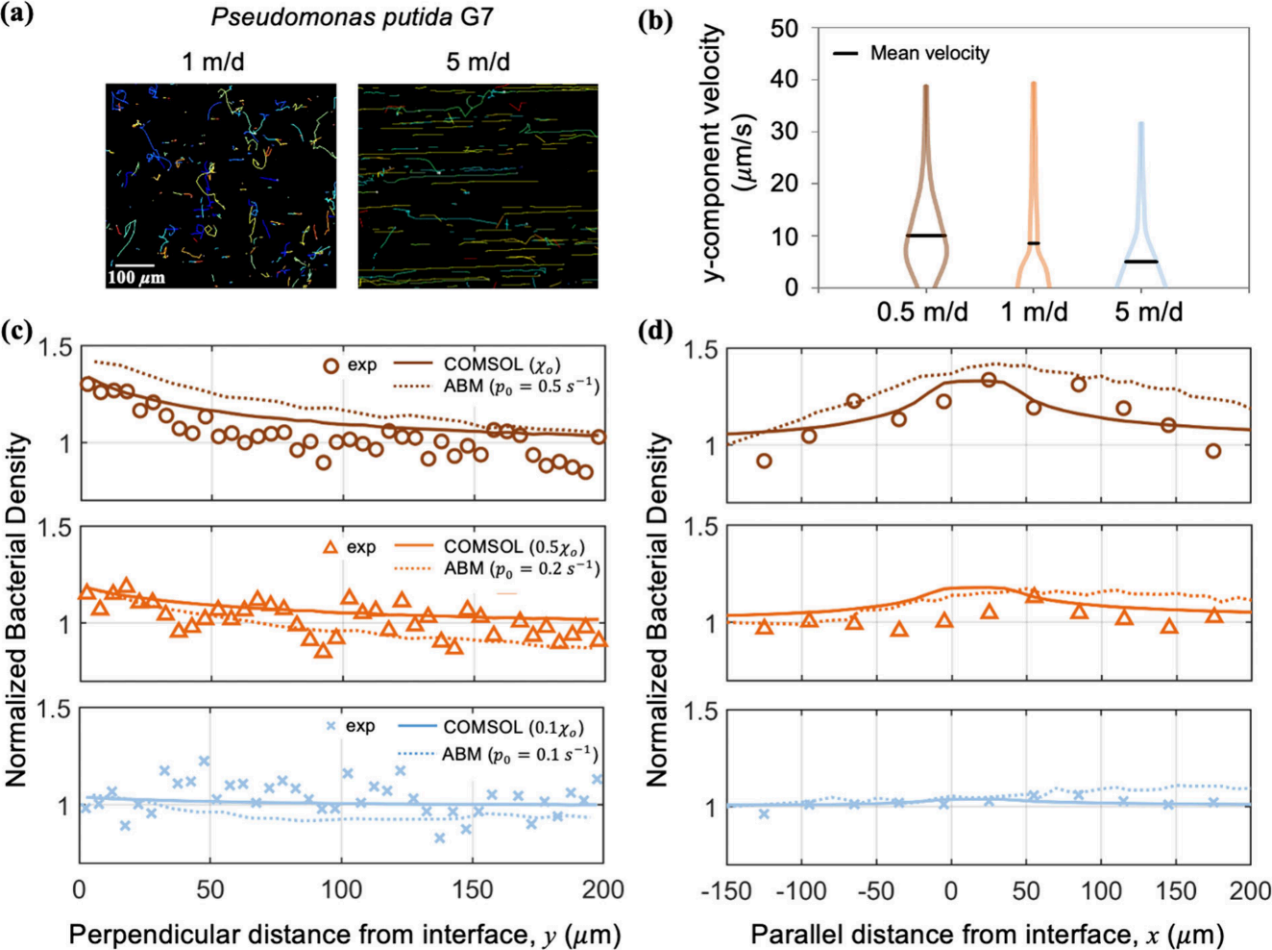
Influence of fluid flow on bacterial swimming properties. (a) *Pp*G7 trajectories at 1 m/d and 5 m/d without chemoattractants. (b) Violin plots for the *y*-component of swimming velocity at 0.5 m/d, 1 m/d, and 5 m/d, with horizontal black lines indicating the average *y*-component values (10.1 μm/s, 8.5 μm/s and 5.0 μm/s, respectively). The width of the violin indicates the relative proportion of bacteria at each velocity along the *y*-axis. Comparison among experimental results (circles, triangles, and crosses), COMSOL simulations (solid lines), and ABM simulations (dotted lines) of bacterial distributions (c) perpendicular and (d) parallel to the NAPL–water interface. Solid lines are continuum model results, also shown in Figure 4.

*P. putida* swim in straight runs that are interrupted by sharp reversals in the swimming direction.^19–21^ To explore how the flow influences their run-reversal pattern and its impact on chemotaxis, we used our agent-based model (ABM) to test the hypothesis that flow modified the reversal frequency (*p*_0_) of *P. putida*, thereby reducing the level of bacterial accumulation near the NAPL–water interface. In the absence of flow, *P. putida* change swimming direction on an average of every two seconds (*p*_0_ = 0.5 s^–1^),^33^ which produced the bacterial profiles at 0.5 m/d in Figure 5c and d for ABM simulations. Reducing *p*_0_ to 0.2 s^–1^ at 1 m/day led to lower bacterial accumulations, consistent with experimental observations. At 5 m/day, a further reduction of *p*_0_ to 0.1 s^–1^ resulted in minimal accumulation near the interface. A parametric analysis of *p*_0_ is provided in the Supporting Information. ABM results provided a mechanistic link between flow-induced suppression of reversals and decreased chemotactic accumulation beyond the effect of convective transport, which is challenging to quantify directly in experiments or the continuum model.

Our ABM allowed us to tune the intrinsic parameters of bacteria and investigate the cellular-level mechanisms by which fluid flow impacts their swimming behaviors and hence their chemotaxis. The continuum model implemented in COMSOL captured macroscopic bacterial density dynamics by reducing chemotactic sensitivity coefficient *χ*_o_ to a different extent at each fluid velocity. According to Rivero et al. (1989),^26^ *χ*_o_ can be expressed as *χ*_o_ = *σN*_T_ *v* ^2^, where σ denotes the change in mean run time per unit time rate of change in receptor occupancy, mathematically $\sigma =\frac{\Delta \tau_{run}}{\Delta \left(\frac{dB}{dt}\right)}$ . If shear flow prevents *P. putida* from reversing, bacteria are increasingly passively advected with a reduced capacity to modulate their directional runs. This leads to less effective chemotactic steering, rendering chemotaxis more asymmetric and less accurate, partially reflected in the reduction of *χ*_*o*_ at the continuum level. However, the continuum model has limitations – it assumes instantaneous response to local gradients and thus cannot capture the flow-induced delays in reorientation or decision-making. Cellular behavior dynamics are better resolved in the ABM framework, where individual cell stochastic decisions and trajectories can be explicitly modeled. The ABM thus provides a mechanistic basis for interpreting how changes in cell-scale parameters such as reversal rate propagate to population-scale patterns.

Bacteria in contaminated subsurface environments typically live in a dynamic world shaped by groundwater flow, which plays a critical role in their transport and the effectiveness of bioremediation. This work examined the chemotaxis of *Pseudomonas putida* toward naphthalene emanating from a NAPL–water interface, which mimics a NAPL droplet trapped within a pore throat in porous media, under fluid velocities representative of natural groundwater conditions (0.5–10 m/d, or shear rates of 0.58 to 11.57 s^–1^) using a T-shaped microfluidic device. The decrease in bacterial accumulation near the NAPL–water interface at higher flow velocities cannot be explained solely by convective flushing according to the continuum-scale simulations. We also explored the influence of convection on bacterial swimming behaviors—specifically, reversal frequency—and the resulting population-level chemotactic response (*χ*_o_). By combining individual- and population-scale modeling, we identified a potential mechanism in which flow-induced suppression of reversal frequency hinders the ability of *P. putida* to navigate chemical gradients. This finding suggests that modulation of reorientation dynamics plays a key role in diminishing chemotactic efficiency at stronger flow conditions. This apparent modification of reversal frequency may result from shear-induced reorientation, such as Jeffery orbits. Future work could further examine this by tracking run-reverse dynamics of *P. putida* to quantify shear-dependent changes in orientation, swimming mode (push or pull), and their influence on chemotaxis.

As bioremediation success relies on effective bacterial targeting of contaminant sources, our findings suggest that chemotactic bacteria were more efficient in locating NAPL contaminant sources at lower groundwater flow rates, where bacterial motility and chemotaxis remain less affected by shear flow. This study integrates experimentation, continuum modeling, and agent-based modeling to bridge individual and population scales of bacterial transport under flow. The presented framework provides a mechanistic basis for understanding how fluid dynamics regulates microbial behavior in complex environments and offers insights for optimizing bioremediation strategies in groundwater systems.

## Supplementary Material



## Data Availability

The Agent-Based Model code is available via GitHub at https://github.com/rhea-braun/pputida-netlogo-Tshaped-shear-ABM.

## Notes

Measurement of naphthalene solubility by gas chromatography, continual model settings and parameters in COMSOL Multiphysics, and Agent-Based modeling parametric analysis are provided in Supporting Information (PDF)
