# Supplementary material for: Pseudomonas putida Chemotactic Efficiency toward Naphthalene at a NAPL–Water Interface Decreased under Increasing Shear Flow
Source: Environ Sci Technol. 2026 Jan 29;60(5):4253–60. doi: 10.1021/acs.est.5c15041 (PMC12895519; doi:10.1021/acs.est.5c15041)
Supplement: Supplementary file 1 [file es5c15041_si_001.pdf]

SUPPORTING INFORMATION

Pseudomonas putida chemotactic efficiency toward naphthalene at a NAPL-water interface decreased under increasing shear flow

Beibei Gao, Rhea Braun, Derek Wu, and Roseanne M. Ford[*]

Department of Chemical Engineering, University of Virginia, Charlottesville, Virginia 22903, United States

*Corresponding authors. Roseanne M. Ford, phone: (+1) 434-924-6283; e-mail: rmf3f@virginia.edu. Mailing address: Department of Chemical Engineering, University of Virginia, 385 McCormick Rd, Charlottesville, VA 22903.

NUMBER OF PAGES: 7

NUMBER OF FIGURES: 5

NUMBER OF TABLES: 1

Gas Chromatography Measurement of Naphthalene Solubility

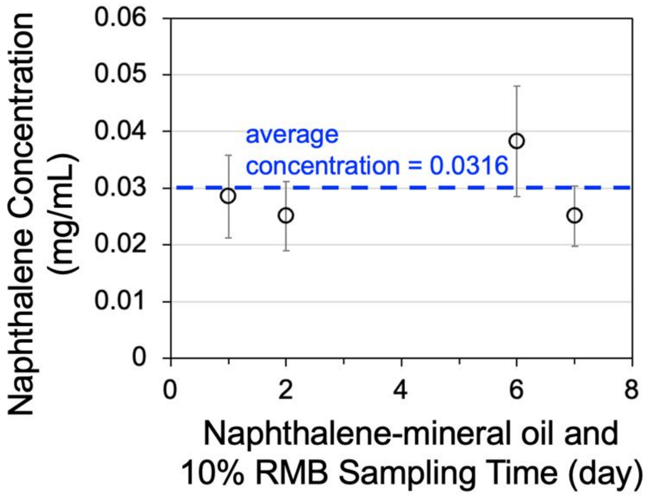

Figure S1. Naphthalene concentration in 10% RMB over time. Error bars represented the standard deviation among three sample replicates. Dashed blue line is the averaged naphthalene concentration, 0.0316 mg/mL, which is 0.247 mol/m^3.

In chemotaxis transport experiments, the side capillary of T-shaped microfluidic chamber was filled with a naphthalene-mineral oil mixture containing 33 g/L naphthalene. To quantify the aqueous-phase solubility of naphthalene partitioned from the oil mixture, we used gas chromatography with a flame ionization detector (GCFID) (GCMS-QP2020 NX (Shimadzu, Germany)). To replicate experimental conditions, 1 mL of the naphthalene-mineral oil mixture was added to 7 mL of 10% Random Motility Buffer (RMB), the same medium used to suspend bacteria in the transport experiments. Aliquots of the aqueous phase were sampled after 1, 2, 6 and 7 days for naphthalene concentration analysis. We used toluene as the organic solvent to extract naphthalene from the aqueous phase, with hexamethylbenzene (HMB) serving as the internal standard for calibration. Figure S1 summarized the naphthalene concentration in 10% RMB across

the sampling period, with an average concentration of 0.0316 mg/mL, corresponding to 0.247 mol/m^3, which is close to naphthalene solubility in water of 0.031 mg/mL at room temperature[1].

Settings and Parameters in COMSOL Multiphysics

Table S1. Parameters used in bacterial chemotaxis simulations.

Parameter	Value	Unit	Reference
Naphthalene diffusion coefficient, D_a	7.5 x 10^{-6}	cm^2/s	(1)
Naphthalene solubility in 10% RMB, a_0	0.25	mM	
Bacterial diffusion coefficient, D_b	3.2 x 10^{-6}	cm^2/s	(2)
Chemotactic sensitivity coefficient (at 0.5 m/d), χ_o	7.2 x 10^{-5}	cm^2/s	(3)
Chemotaxis receptor constant, K_c	0.016	mM	(3)
*Pp*G7 swimming speed, *v*	49	μm/s	(3)
Initial bacterial density (normalized), b_0	1	[-]	

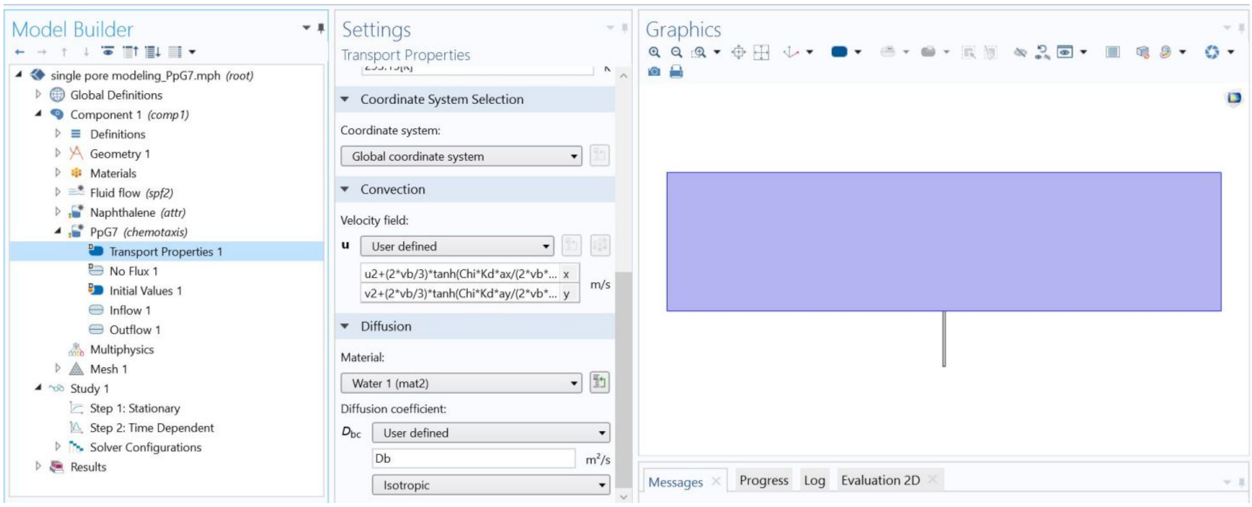

Figure S2. Screenshot of COMSOL Multiphysics interface showing the domain and modules used in our simulations.

Continuum models for chemoattractant and bacteria were solved using COMSOL Multiphysics version 5.6 Transport of Diluted Species module. Figure S2 shows the interface of COMSOL and the implementation of our model. Parameters and their values are listed in Table

S1. The microfluidic chamber has a width of 2.5 mm and a height of 20 μm, resulting in dominant shear gradients in the vertical (z) direction. We tested the influence of shear in the x-y plane near the NAPL-water interface by applying No-slip and Slip boundary conditions at all walls except the inlet and outlet (Figure S3). Our simulations showed that shear in the x-y plane did not reproduce the reduced chemotactic accumulation observed experimentally.

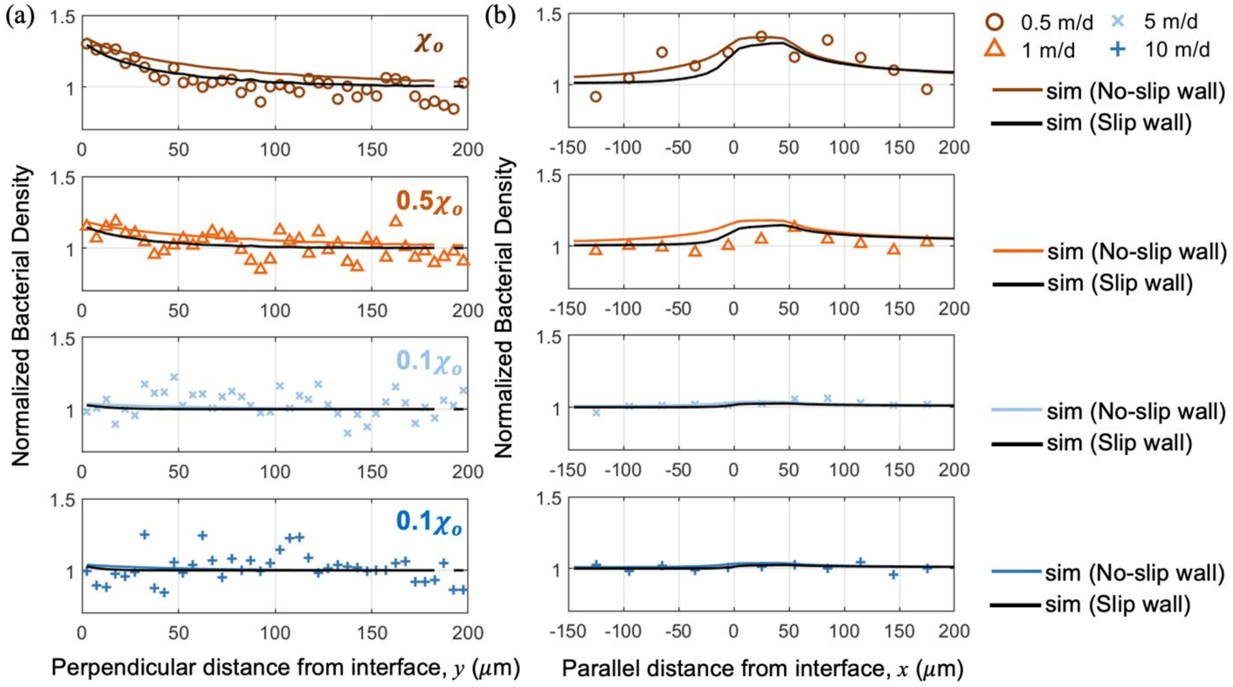

Figure S3. A comparison of continuum simulations at No-slip (colored solid lines) and Slip (black solid lines) boundary conditions. Symbols are experimental results. Chemotactic sensitivity coefficient, χ_o = 7.2×10^{-5} cm^2/s.

Agent-Based Modeling Parametric Analysis

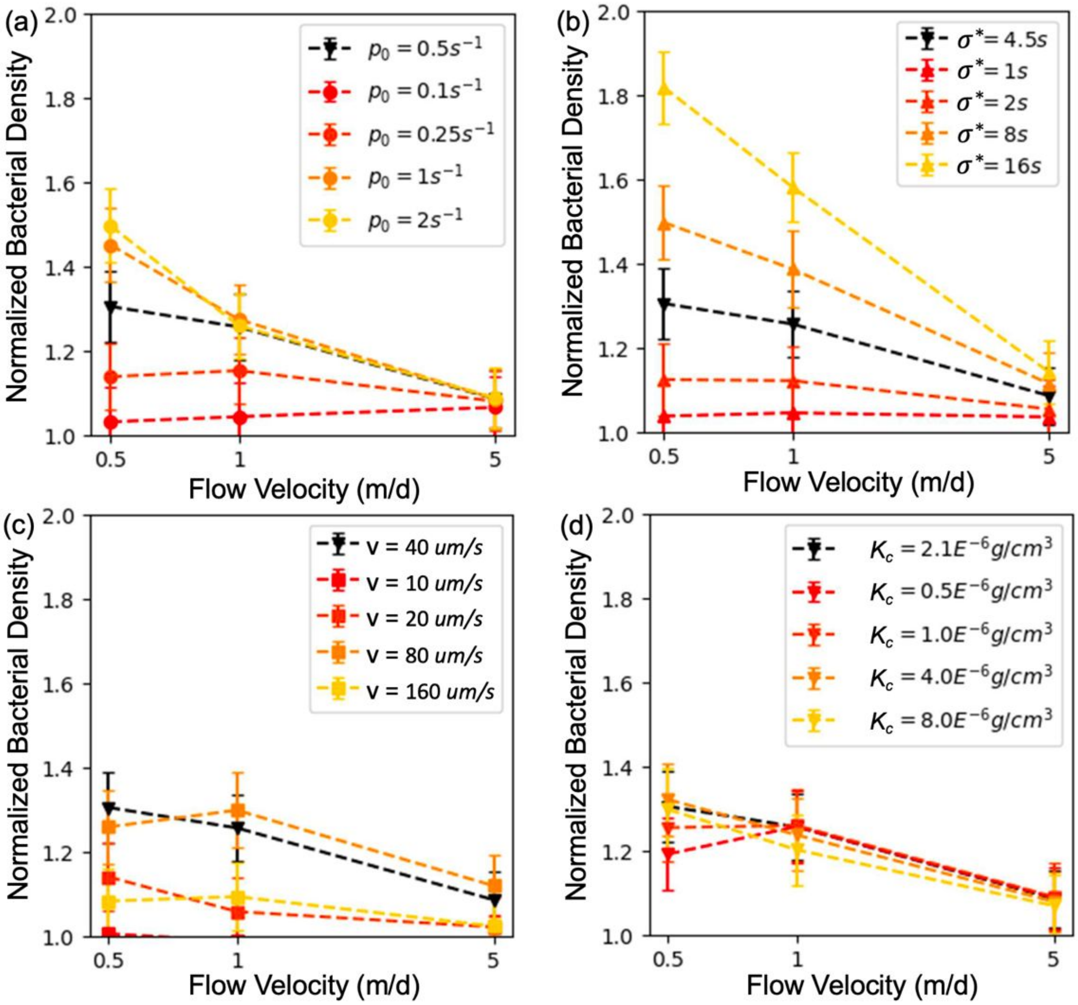

Figure S4. Sensitivity analysis of ABM parameters p_0, σ^*, v and K_C at 0.25, 0.5, 1, and 2 times (red to yellow lines) the baseline value (black lines) showing the average accumulation at the NAPL-water interface for the three fluid velocities (0.5, 1, and 5 m/d).

In the main text, the tumbling probability of a cell undergoing chemotaxis is defined as,

$$\log\left(\frac{p_t^+}{p_0}\right) = -\,\sigma^* \frac{K_C}{(K_C + a)^2}\ \frac{Da}{Dt} \tag{1}$$

In the Agent-Based Model (ABM), we varied the baseline reversal rate p_0, effective single-cell chemotactic sensitivity σ^*, bacterial swimming speed v, and receptor constant K_C to investigate the influence of intrinsic cellular parameters on bacterial distribution. As shown in Figure S4,

bacterial accumulation decreased with lower values of p_0 and σ^*. Further analysis of bacterial distributions along x and y directions revealed that changes in reversal rate most effectively explained the observed variations in bacterial accumulation across different flow velocities, as described Figure S5 and in detail in the main text. In Figure S4c and d, for a given flow velocity the variations in the swimming speed and receptor constant produced more complex, non-monotonic effects on bacterial accumulation. We cannot rule out the changes in swimming speed or receptor constant without direct measures on them, however, there is no experimental evidence that these two factors intrinsically change under shear flow. Please note that COMSOL results in Figure S5 (solid lines) were obtained from simulations without adjusting the chemotactic sensitivity coefficient χ_o across different fluid velocities.

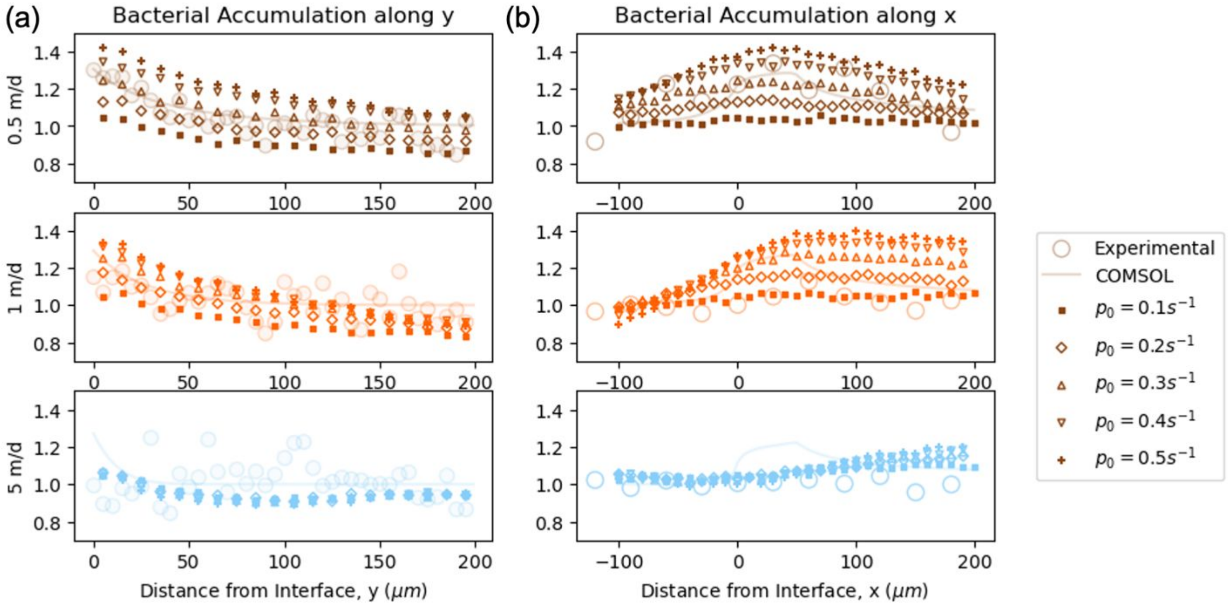

Figure S5. Sensitivity analysis of reversal frequency p_0 at each fluid flow velocity.

REFERENCES

(1) Gustafson, K. E.; Dickhut, R. M. Molecular Diffusivity of Polycyclic Aromatic Hydrocarbons in Aqueous Solution. *J. Chem. Eng. Data* **1994**, *39* (2), 281–285. https://doi.org/10.1021/je00014a019.

(2) Marcos; Fu, H. C.; Powers, T. R.; Stocker, R. Bacterial Rheotaxis. *Proc. Natl. Acad. Sci. U. S. A.* **2012**, *109* (13), 4780–4785. https://doi.org/10.1073/pnas.1120955109.

(3) Marx, R. B.; Aitken, M. D. Quantification of Chemotaxis to Naphthalene by Pseudomonas Putida G7. *Appl. Environ. Microbiol.* **1999**, *65* (7), 2847–2852. https://doi.org/10.1128/aem.65.7.2847-2852.1999.

(4) Wang, X.; Long, T.; Ford, R. M. Bacterial Chemotaxis toward a NAPL Source within a Pore-Scale Microfluidic Chamber. *Biotechnol. Bioeng.* **2012**, *109* (7), 1622–1628. https://doi.org/10.1002/bit.24437.
